# Sociodemographic and clinical factors related to high rates of unmet supportive care needs among Polish cancer patients

**DOI:** 10.3389/fpsyg.2025.1472026

**Published:** 2025-05-14

**Authors:** Karolina Osowiecka, Marek Szwiec, Jacek J. Nowakowski, Marcin Kurowicki, Anna Dolińska, Anna Gwara, Eliza Działach, Jarosław Kołb-Sielecki, Weronika Radecka, Sergiusz Nawrocki, Monika Rucińska

**Affiliations:** ^1^Department of Psychology and Sociology of Health and Public Health, School of Public Health, University of Warmia and Mazury in Olsztyn, Olsztyn, Poland; ^2^Department of Surgery and Oncology, Faculty of Medicine and Health Sciences, University of Zielona Gora, Zielona Góra, Poland; ^3^Department of Botany and Evolutionary Ecology, Faculty of Biology and Biotechnology, University of Warmia and Mazury in Olsztyn, Olsztyn, Poland; ^4^NU-MED Radiotherapy and Oncology Center in Elbląg, Elbląg, Poland; ^5^Psychology Outpatient Clinic, University Hospital in Zielona Gora, Zielona Góra, Poland; ^6^Department of Nursing, Institute of Health Science, University of Zielona Gora, Zielona Góra, Poland; ^7^Department of Public Health, Faculty of Sciences in Bytom, Medical University of Silesia in Katowice, Katowice, Poland; ^8^Department of Oncology, The Center for Pulmonary Diseases in Olsztyn, Olsztyn, Poland; ^9^Department of Anatomy, University of Opole, Opole, Poland; ^10^Department of Oncology, Collegium Medicum, University of Warmia and Mazury in Olsztyn, Olsztyn, Poland

**Keywords:** cancer, NEQ, unmet needs, supportive care, total and subtotal NEQ scores

## Abstract

**Introduction:**

Cancer patients experience a wide range of unmet supportive care needs, which may lead to dissatisfaction with the health care system and poor quality of life. The aims of this study were to assess unmet supportive care needs in five areas: informative needs, psycho-emotional needs, relational needs, material needs and needs related to assistance/care, and to identify groups of cancer patients expressing high rates of unmet needs.

**Materials and methods:**

The multicenter study was carried out among 1,062 cancer patients between June 2022 and May 2023. The study was performed using the validated Polish version of Needs Evaluation Questionnaire (NEQ). The total and subtotal NEQ scores were calculated.

**Results:**

The mean total NEQ score was 0.44 ± 0.27. The highest subtotal NEQ scores were noted in the cases of informative needs and material needs. Predictive factors for a higher total NEQ score were younger age, living with a partner and a diagnosis of digestive system cancers or lung cancer.

**Conclusion:**

Cancer patients expressed some unmet supportive care needs, especially informative needs. Identification of the groups of patients with high rates of unmet supportive care needs will be useful in clinical practice. Younger patients, living with a partner and those suffering from digestive system or lung cancer seem to require special attention.

## 1 Introduction

Cancer is a worldwide problem, not only medically, but also socio-psychologically, as it is a particularly destructive disease for patients. Cancer patients experience a broad variety of problems including difficulties with physical functioning, an inability to work, loss of previous social role, marital distress, breakdown of social relationships, sexual dysfunction, daily living functioning (Sanson-Fisher et al., 2000; Malone et al., 1994; Graf et al., 2020; Harrison et al., 2009). Cancer diagnosis can lead to disease-related distress, anxiety, hopelessness, various psychological morbidities (Ahmad et al., 2015; Mehnert et al., 2014; Mielcarek et al., 2016; Zabora et al., 2001), and decreased quality of life (Lewandowska et al., 2020a; Alam et al., 2020; Okediji et al., 2017). There is a potential gap between patients’ psycho-social expectations and oncological health care system service. Deficiencies in non-medical supportive care may lead to cancer patients’ dissatisfaction with treatment (Okediji et al., 2017). Cancer patients experience a wide range of unmet non-medical/supportive care needs (Okediji et al., 2017; Tamburini et al., 2003; Sutherland et al., 2009; Davis et al., 2003; Nixon and Narayanasamy, 2010). Fitch (2000) defined unmet supportive care needs as a lack of support that an individual perceives as necessary to reach the best possible well-being. Unmet needs have been categorized into specific areas: informational, emotional, psycho-social, spiritual, physical and practical (Graf et al., 2020; Evans Webb et al., 2021; Annunziata et al., 2009). Unmet supportive care needs may lead to an inability to cope effectively with distress related to illness and a poorer quality of life (Hack et al., 2010; Fitch and Maamoun, 2016; Park and Hwang, 2012; Cochrane et al., 2022). Bonacchi et al. (2019) showed a positive association between psychological distress and a higher level of unmet needs across all need areas. Uslu-Sahan and Gulcan (2023) observed that the acceptance of cancer was negatively correlated with unmet supportive care needs. It was noted that unmet non-medical needs of cancer patients were negatively correlated with a sense of hope (Ripamonti et al., 2016). Anxiety and depression were also associated with patients’ unmet needs (Erdoğan Yüce et al., 2021; Bunston et al., 1998; Boyes et al., 2012; Akechi et al., 2011; Chambers et al., 2012; Dyson et al., 2012).

There are a variety of supportive care needs related to cancer. Each individual patients will have their own specific needs. Supportive care needs may be influenced by type of cancer, clinical stage, treatment modality and some sociodemographic factors (for example age, sex, marital status, financial status) (Sanson-Fisher et al., 2000; Okediji et al., 2017; Bonacchi et al., 2019; Puts et al., 2012). In Poland, there is a lack of studies on assessing the unmet non-medical needs among cancer patients. To identify supportive care needs some instruments have been designed (Rimmer et al., 2022; Wen and Gustafson, 2004; Richardson et al., 2007; Cassileth et al., 1980). The use of surveys to self-report may help patients to express their expectations. On the other hand, medical staff could more easily identify and meet these needs. Among the instruments available to assess patients’ unmet needs, the Needs Evaluation Questionnaire (NEQ) (Tamburini et al., 2003; Tamburini et al., 2000) seems to be an effective tool. The NEQ is simple, consists of 23 dichotomous questions and takes about 10 min to complete (Tamburini et al., 2000; Bonacchi et al., 2016; Osowiecka et al., 2024). The NEQ is the first validated questionnaire to assess non-medical needs among Polish cancer patients (Osowiecka et al., 2024). The identification of unmet supportive care needs should be a part of cancer policy. It is crucial to identify the individual patients’ real needs and to recognize groups of patients with a higher probability of unmet supportive care needs. Awareness about patients’ unmet needs and concerns is important for planning some interventions which may reduce stress among cancer patients (Bonacchi et al., 2010) and improve patients’ quality of life (Cochrane et al., 2022).

NEQ addresses needs in five domains: informative needs, psycho-emotional needs, relational needs, material needs and needs related to assistance/care (Annunziata et al., 2009). Information needs seems to be the most frequently unmet among cancer patients undergoing oncological therapy (Okediji et al., 2017; Evans Webb et al., 2021). Patients have expressed a need for different types of information related to their disease, examination, treatment options, side effects of treatment and prognosis (Cassileth et al., 1980; Neumann et al., 2011; Fallowfield et al., 1995). Evans Webb et al. (2021) conducted a review including 32 worldwide studies and proved that cancer patients were more likely to receive information about what treatment they would receive, why that treatment was proposed, how it would work, what the likelihood of a cure is, what they can expect from their disease and therapy, how long they would stay in hospital, and when they would return to “normal life” (Evans Webb et al., 2021). The awareness about what information related to disease and treatment patients would like to receive from their oncologists could improve patient-doctor communication (Ha and Longnecker, 2010). Effective identification of information needs seems to help improve a patient’s perception of the disease, treatment decision, understanding doctor’s instructions, compliance with treatment, perception of the future, quality of life, and even treatment outcomes (Sobczak et al., 2018; Zolnierek and Dimatteo, 2009; Mager and Andrykowski, 2002). Neumann et al. (2011) reported that a good patient-doctor relationship (patient’s perception of a high degree of physician empathy) was the most significant predictor of well-met information needs.

Cancer patients have expressed unmet psycho-emotional needs (Driessen et al., 2023; Amenu et al., 2023). Some studies (Tamburini et al., 2003; Bonacchi et al., 2016) reported that cancer patients had a need to speak with people who have had the same experience, to speak with a psychologist and to speak with spiritual assistant. Other study (Neumann et al., 2011) showed that cancer patients wanted information about psychological/psychotherapeutic support (31%) and self-help groups (20%). Giuliani et al. (2016) reported a relatively high level of psychological unmet needs among lung cancer patients. The most frequently indicated unmet needs were “fears of the cancer spreading” and “uncertainty about the future.” Among young adult cancer patients in Japan, psychological needs were the most reported unmet supportive care needs (“fears of spreading of cancer,” “feeling down or depressed” and “anxiety”) (Okamura et al., 2021). Our previous study (Osowiecka et al., 2020) showed that a lot of Polish cancer patients also had this kind of needs. However they received psychological support mainly from family/friends and only 21% of patients got support from psychologist and 4% of them from a priest (Osowiecka et al., 2020). The unmet psychological needs may have the impact on quality of life and higher psychological distress (Cochrane et al., 2022).

Unmet relational needs were also reported among cancer patients. Patients needed to feel more useful in their family, to be more reassured by their relatives and to feel less abandoned (Tamburini et al., 2003; Bonacchi et al., 2016). However, patients needed to be less commiserated by others (Tamburini et al., 2003; Bonacchi et al., 2016). The commiseration from other people was considered as “something negative” and “excessive pitying of someone” by most of previously investigated cancer patients (Osowiecka et al., 2024).

Oncological therapy causes some material needs and need for economic help (Tamburini et al., 2003; Bonacchi et al., 2016). Cancer patients were likely to receive more information regarding social issues how to solve employment legislation problems, health insurance problems, financial problems, etc. (Tamburini et al., 2003; Bonacchi et al., 2016; Neumann et al., 2011; Driessen et al., 2023). In previous study (Osowiecka et al., 2020) only 7% of cancer patients received a sufficient support from a social worker.

It seems that needs related to assistance/care were well met. However some patients needed more support in daily activities, more attention from nurses and more respect for their intimacy (Graf et al., 2020; Tamburini et al., 2003; Bonacchi et al., 2016; Giuliani et al., 2016; Sodergren et al., 2019).The aims of this study were to assess unmet supportive care needs in all those five areas: informative needs, psycho-emotional needs, relational needs, material needs and needs related to assistance/care, and to identify groups of cancer patients expressing high rates of unmet needs.

## 2 Materials and methods

### 2.1 The study group and design

#### 2.1.1 Patients

The study was conducted between June 2022 and May 2023 on a group of 1,062 consecutive patients, who were treated for a variety of cancer types in seven oncological centers located in different regions of Poland (Hospital of the Ministry of Internal Affairs with Warmia and Mazury Oncology Center in Olsztyn, The Center for Pulmonary Diseases in Olsztyn, NU-MED Radiotherapy Center in Elblag, Hospital in Prabuty, Oncology Center in Opole, Zaglebiowskie Oncology Center in Dabrowa Gornicza, University Hospital in Zielona Gora).

#### 2.1.2 Sample

The sample size was calculated to be representative for this cross-sectional study (Tabachnick and Fidell, 2007). One thousand hundred and eighty cancer patients met the inclusion criteria: 18 years or older, histopathological confirmation of cancer diagnosis, oncology treatment ongoing or patient having finished treatment no longer than 3 months previously, current hospitalization for at least 3 days or at least one hospitalization due to oncological treatment within the previous 3 months. One thousand sixty-two patients (90%) decided to participate in the study.

### 2.2 Questionnaire

**Figure 1:**
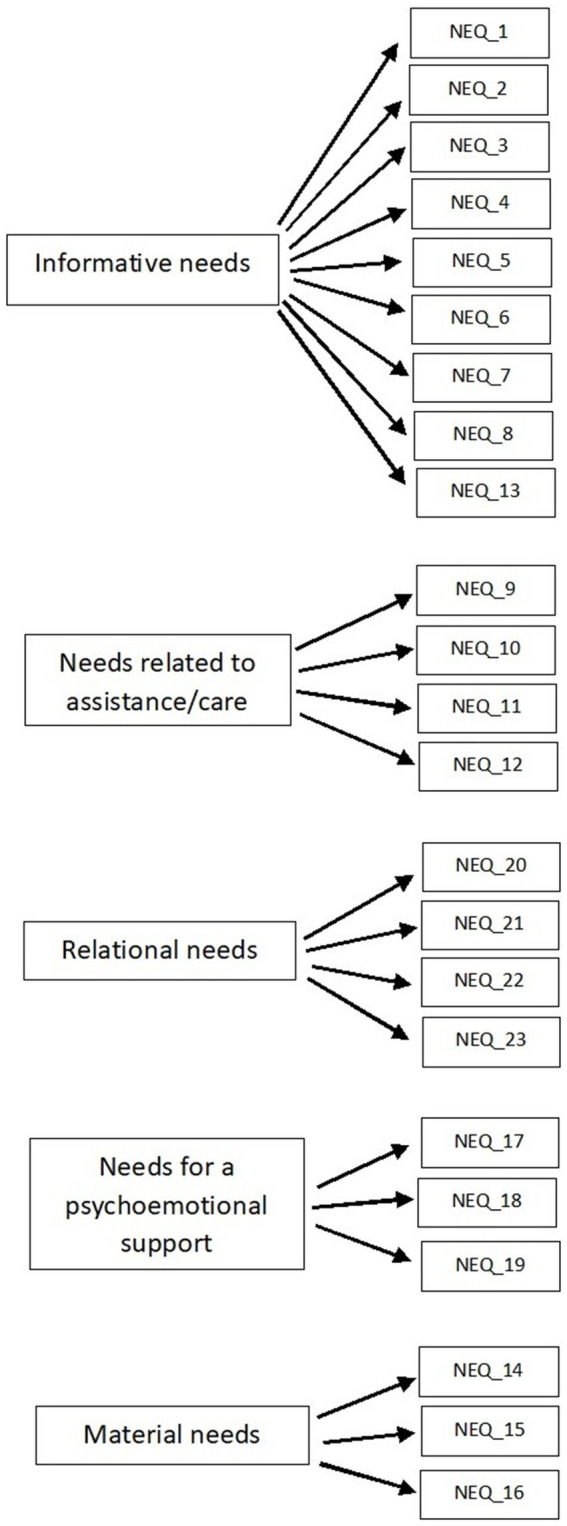
The five-factor model of the NEQ modified based on Annunziata et al., 2009.

The study was performed using the Needs Evaluation Questionnaire (NEQ). The NEQ is a self-administered, comprehensive questionnaire that consists of 23 items with responses on a dichotomous scale (yes/no). The NEQ was originally designed and validated among Italian cancer patients (Tamburini et al., 2003; Tamburini et al., 2000). Permission for use of the NEQ in a Polish population was obtained from the authors. According to Annunziata’s distinction (Annunziata et al., 2009) the NEQ was used to assess patients’ needs in five areas (Figure 1).

#### 2.2.1 Questionnaire validity and reliability

The psychometric properties of the questionnaire were investigated (Osowiecka et al., 2024). The questionnaire showed good reliability and internal factor structure validity. Confirmatory factor analysis of the selected model showed a good overall fit for the model (χ2 = 518.37; *p* < 0.001). The values of the estimated fit indices (CFI = 0.801; PNFI = 0.612; SRMR = 0.077) were very close to the values considered to be satisfactory for model fit. All estimated standardized factor loadings ranged from 0.52 to 0.84 and were significantly different from zero at *p* < 0.001, confirming good levels of internal factor structure validity. In general, inter-correlations between pairs of distinguished groups were very high (>0.7). Only the levels of inter-correlations between informative needs and relational needs and between informative needs and need for psycho-emotional support were close to 0.7. Cronbach’s *α* indexes for the five factors were ≥0.7. Test–retest reliability coefficients have ranged from 0.60 to 0.95 over a 2-week interval (Osowiecka et al., 2024).

The structure, length and font size of the NEQ were acceptable to respondents. The meanings of the questions were well understood (Osowiecka et al., 2024).

The NEQ was supplemented by questions concerning sociodemographic and clinical data (age, gender, educational level, place of residence, employment, marital status, household, having a physician among family/friends, type of cancer and approximate date of cancer diagnosis). Polish and English versions of the NEQ are presented in Supplementary materials.

#### 2.2.2 Data collection

Patients undergoing oncological therapy (in-and out-patients) were proposed to taking part in the study. Data was collected using a paper questionnaire with eventual help from psychologist or nurse.

#### 2.2.3 Ethical agreement

The study protocol was approved by the Ethics Committee of the University of Warmia and Mazury in Olsztyn (No. 30/2020). Participation in the study was voluntary. All study participants were informed about the aim of the study and gave their signed consent.

### 2.3 Statistical analysis

The total NEQ score was calculated by summing the total number of unmet needs divided by the number of 23 NEQ items for each patient. Scores for each area of needs were calculated by summing the number of unmet needs in each area divided by the number of needs in that area (informative needs: 9, assistance/care needs: 4, relational needs: 4, psycho-emotional support needs: 3 and material needs: 3). The score for all and each need ranges from 0 to 1. The mean total and subtotal NEQ scores were calculated. The differences between NEQ scores were determined using one-way ANOVA analysis with Tukey’s test of multiple comparisons. Intercorrelations were estimated using partial correlation coefficient. Partial correlations allow for the assessment of the linear degree of association between variables, taking into account the influence of one or more additional variables. The partial correlation coefficient then informs us about the independent influence of a given variable on the dependent variable (when the influence of other variables is eliminated). In contrast to interdependent equations models, recursive models impose constraints on the construction of the equations appearing in the model and if the model is recursive, then only one-way relationships exist between variables. Generalized linear models (GLZ), with the Akaike information criterion (AIC) for normal distribution and identity function, were used to determine the relationship between the supportive care needs and a set of independent variables (age, gender, educational level, place of residence, employment, marital status, household, having a physician among family/friends, type of cancer and time from cancer diagnosis). The best fit GLZ have been presented in this analysis. A *p*-value of < 0.05 was considered to be significant. The data analysis was conducted using Statistica (data analysis software), version 13. http://statistica.io TIBCO Software Inc., Krakow, Poland (2017) and IBM SPSS Statistics 29.0 software (IBM Corp, 2020).

## 3 Results

### 3.1 Characteristics of patients

The study was carried out on a group of 1,062 cancer patients: 52% men and 48% women. Respondents were 22–89 years old (median age 66 years). The majority of patients: lived in cities (63.6%), had graduated from secondary school (68.1%), were pensioners (73.4%), married (67.8%), and living with a partner (66.5%). 15.1% of respondents had a physician in their close family or among friends. Lung cancer, lower digestive system cancers and breast cancer were the most frequent (28.2, 19.5, 15.6%, respectively). The median time from cancer diagnosis was 6 months (Table 1).

**Table 1:** Study group demographics.

Variables	*n*	%
Age: median 66 years (range 22–89 years)		
Gender		
Female	507	47.7
Male	553	52.1
No data	2	0.2
Place of residence		
City	676	63.6
Village	383	36.1
No data	3	0.3
Marital status		
Married	720	67.8
Relationship broken down during disease or in relation to disease	5	0.5
Single	332	31.2
No data	5	0.5
Household		
With partner	706	66.5
With child/children and/or another family member	166	15.6
Alone	171	16.1
No data	19	1.8
Educational level		
Primary	180	17.0
Secondary	723	68.1
High	147	13.8
No data	12	1.1
Employment		
Employed	236	22.2
Unemployed	41	3.9
Pensioner	780	73.4
No data	5	0.5
Physician as a member of family or friends		
Yes	160	15.1
No	893	84.1
No data	9	0.8
Localization of cancer		
Head and neck	62	5.9
Upper digestive system	71	6.7
Lower digestive system	207	19.5
Lung	300	28.2
Breast	166	15.6
Gynecological	55	5.2
Prostate	109	10.3
Brain	15	1.4
Urinary system	30	2.8
Other	31	2.9
No data	16	1.5

Time from cancer diagnosis: median 6 months (range 1–265 months).

### 3.2 Needs prevalence

The mean total NEQ score was 0.44 ± 0.27. The highest subtotal NEQ scores were noted in the cases of informative needs (mean 0.55 ± 0.37) and material needs (mean 0.49 ± 0.35). Lower subtotal NEQ scores were reported for relational needs (mean 0.40 ± 0.36), needs related to assistance/care (mean 0.30 ± 0.31) and needs for psycho-emotional support (mean 0.28 ± 0.33). The differences between all subtotal NEQ scores for unmet supportive care needs were significant (Tukey’s test: *p* < 0.001), except for a pair of needs for psycho-emotional support and needs related to assistance/care (*p* = 0.62). Significant positive correlations were estimated between some of the analyzed domains of all five domains of need (*p* < 0.05), with partial correlation coefficients indicating a weak association between the variables (Table 2). The frequency of needs in each domain was presented in Table 3.

**Table 2:** Partial correlation coefficients between five need domains.

Dimension	Informative needs	Needs related to assistance/care	Relational needs	Needs for a psychoemotional support	Material needs
Informative needs	–				
Needs related to assistance/care	0.418*	–			
Relational needs	0.207*	0.235*	–		
Needs for a psycho-emotional support	0.004	0.157*	0.297*	–	
Material needs	0.172*	0.182*	0.080	0.201*	–

*Estimated correlations significant at the *p* < 0.001 level.

**Table 3:** Frequency of needs per domain.

Dimension	Item number		Yes		No		No data	
			*n*	%	*n*	%	*n*	%
Informative needs	Q1	I need more information about my diagnosis	531	50.0	525	49.4	6	0.6
	Q2	I need more information about my future condition	729	68.7	321	30.2	12	1.1
	Q3	I need more information about the exams I am undergoing	580	54.6	463	43.6	19	1.8
	Q4	I need more explanations of treatments	636	59.9	405	38.1	21	2.0
	Q5	I need to be more involved in the therapeutic choices	496	46.7	532	50.1	34	3.2
	Q6	I need clinicians and nurses to give me more comprehensible information	555	52.3	489	46.0	18	1.7
	Q7	I need clinicians to be more sincere with me	608	57.3	437	41.1	17	1.6
	Q8	I need to have a better dialogue with clinicians	508	47.8	535	50.4	19	1.8
	Q13	I need to be more reassured by the clinicians	614	57.8	422	39.7	26	2.5
Needs related to assistance/care	Q9	I need my symptoms (pain, nausea, insomnia, etc.) to be better controlled	545	51.3	500	47.1	17	1.6
	Q10	I need more help with eating, dressing, and going to the bathroom	132	12.4	914	86.1	16	1.5
	Q11	I need better respect for my intimacy	296	27.9	746	70.2	20	1.9
	Q12	I need better attention from nurses	311	29.3	728	68.5	23	2.2
Material needs	Q14	I need better services from the hospital (bathrooms, meals, cleaning)	590	55.6	438	41.2	34	3.2
	Q15	I need to have more economic insurance information (tickets, invalidity, etc.) in relation to my illness	644	60.7	390	36.7	28	2.6
	Q16	I need economic help	330	31.1	702	66.1	30	2.8
Needs for a psycho-emotional support	Q17	I need to speak with a psychologist	254	23.9	774	72.9	34	3.2
	Q18	I need to speak with a spiritual advisor	216	20.3	813	76.6	33	3.1
	Q19	I need to speak with people who have this same experience	426	40.1	609	57.3	27	2.6
Relational needs	Q20	I need to be more reassured by my relatives	389	36.6	640	60.3	33	3.1
	Q21	I need to feel more useful within my family	567	53.4	468	44.1	27	2.5
	Q22	I need to feel less abandoned	364	34.3	664	62.5	34	3.2
	Q23	I need to receive less commiseration from other people	377	35.5	663	62.4	22	2.1

### 3.3 Association between unmet supportive care needs and sociodemographic and clinical factors

**Figure 2:**
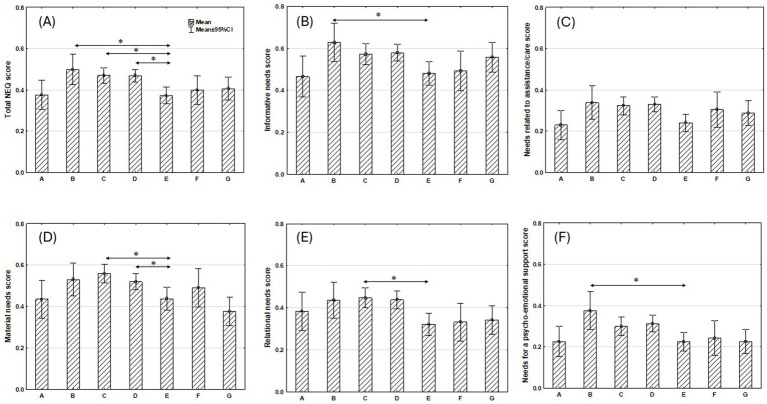
Mean of total and subtotal NEQ scores due to cancer localization: **(A)** head and neck cancer, **(B)** upper digestive system cancer, **(C)** lower digestive system cancer, **(D)** lung cancer, **(E)** breast cancer, **(F)** gynecological cancer, **(G)** prostate cancer. *Significant differences between subgroups based on GLZ were presented in Tables 4–9.

In the multivariate model the total NEQ score was significantly associated with age, household and cancer localization. The total NEQ score decreased with age (*p* = 0.009). A higher total NEQ score was observed among patients living with a partner than those living alone (*p* = 0.03). The highest total NEQ scores were noted in patients diagnosed with upper digestive system cancers, lower digestive system cancers and lung cancer in comparison with the lowest total NEQ score noted among breast cancer patients (respectively, *p* = 0.016, *p* = 0.011 and *p* = 0.007) (Table 4 and Figure 2).

**Table 4:** Generalized linear models (GLZ) to explain the relationship between the total NEQ and a set of independent variables.

Effect	Level of effect	Parameter	95% CI of parameter	Wald function	*p*
Age		−0.002	−0.004 – −0.001	6.739	0.009
Household (Ref.: alone)	With partner	0.029	0.004–0.054	5.016	0.025
	With child/children and/or another family member	0.004	−0.029 – 0.037	0.051	0.822
Localization of cancer (Ref.: breast)	Head and neck	−0.061	−0.122 – 0.001	3.762	0.052
	Upper digestive system	0.072	0.013–0.130	5.754	0.016
	Lower digestive system	0.049	0.011–0.087	6.510	0.011
	Lung	0.046	0.012–0.080	7.185	0.007
	Gynecological	−0.040	−0.105 – 0.024	1.493	0.222
	Prostate	−0.005	−0.054 – 0.044	0.044	0.834

AIC = 216.18; maximum likelihood estimation chi^2^ = 37.53; link function: identity; *p* < 0.001.

There was a negative correlation between informative needs score and age (p = 0.03). Higher informative needs scores were reported by married patients than those who were single (*p* = 0.003) and upper digestive system cancer patients in comparison with breast cancer patients (*p* = 0.02) (Table 5 and Figure 2).

**Table 5:** Generalized linear models (GLZ) to explain the relationship between the informative needs and a set of independent variables.

Effect	Level of effect	Parameter	95% CI of parameter	Wald function	*p*
Age		−0.003	−0.005 – −0.001	4.525	0.033
Marital status (Ref.: single)	Married	0.038	0.013–0.064	8.716	0.003
Physician as a member of family or friends (Ref.: yes)	No	0.024	−0.008 – 0.057	2.136	0.144
Localization of cancer (Ref.: breast)	Head and neck	−0.083	−0.166 – − 0.001	3.906	0.048
	Upper digestive system	0.092	0.013–0.170	5.176	0.023
	Lower digestive system	0.044	−0.007 – 0.094	2.812	0.094
	Lung	0.044	−0.002 – 0.089	3.576	0.059
	Gynecological	−0.060	−0.147 – 0.028	1.803	0.179
	Prostate	0.024	−0.043 – 0.090	0.494	0.482

AIC = 769.9; maximum likelihood estimation chi^2^ = 31.1; link function: identity; *p* < 0.001.

With age, a significant decrease in the assistance/care needs score was observed (*p* = 0.001). Higher scores for needs related to assistance/care were noted among males than females (*p* = 0.006) and patients with a lower educational level compared to those with a higher education (*p* = 0.048) (Table 6 and Figure 2).

**Table 6:** Generalized linear models (GLZ) to explain the relationship between the needs related to assistance/care and a set of independent variables.

Effect	Level of effect	Parameter	95% CI of parameter	Wald function	*p*
Age		−0.004	−0.007 - − 0.002	10.639	0.001
Gender (Ref.: male)	Female	−0.028	−0.049 - − 0.008	7.608	0.006
Educational level (Ref.: high)	Primary	0.038	0.001–0.076	3.912	0.048
	Secondary	−0.024	−0.052 – 0.004	2.735	0.098
Employment (Ref.: pensioner)	Employed	−0.009	−0.055 – 0.037	0.148	0.701
	Unemployed	−0.050	−0.121 – 0.020	1.936	0.164

AIC = 434.22; maximum likelihood estimation chi^2^ = 19.10; link function: identity; *p* = 0.004.

Younger age was a predictor for a higher score of material needs (*p* < 0.001). Higher material needs scores were reported by pensioners than professionally active patients (*p* = 0.01) and patients diagnosed with lower digestive system cancer and lung cancer in comparison with breast cancer patients (respectively, *p* < 0.001 and *p* = 0.01) (Table 7 and Figure 2).

**Table 7:** Generalized linear models (GLZ) to explain the relationship between the material needs and a set of independent variables.

Effect	Level of effect	Parameter	95% CI of parameter	Wald function	p
Age		−0.007	−0.010 – −0.004	20.296	<0.001
Gender (Ref.: male)	Female	0.021	−0.006 – 0.049	2.323	0.128
Place of residence (Ref.: village)	City	−0.021	−0.043 – 0.002	3.150	0.076
Employment (Ref.: pensioner)	Employed	−0.065	−0.116 – 0.014	6.263	0.012
	Unemployed	0.070	−0.008 – 0.148	3.066	0.080
Localization of cancer (Ref.: breast)	Head and neck	−0.05	−0.125 – 0.031	1.418	0.234
	Upper digestive system	0.064	−0.011 – 0.138	2.830	0.093
	Lower digestive system	0.091	0.043–0.138	13.742	<0.001
	Lung	0.053	0.011–0.095	6.069	0.014
	Gynecological	−0.051	−0.137 – 0.035	1.361	0.244
	prostate	−0.035	−0.102 – 0.031	1.075	0.299

AIC = 636.27; maximum likelihood estimation chi^2^ = 62.88; link function: identity; *p* < 0.001.

Higher relational needs scores were expressed by patients with a lower education level in comparison with those who graduated with a higher level (*p* = 0.004) and lower digestive system cancer patients than breast cancer patients (*p* = 0.006) (Table 8 and Figure 2).

**Table 8:** Generalized linear models (GLZ) to explain the relationship between the relational needs and a set of independent variables.

Effect	Level of effect	Parameter	95% CI of parameter	Wald function	*p*
Age		−0.002	−0.005 – − 0.001	3.107	0.078
Educational level (Ref.: high)	Primary	0.068	0.022–0.113	8.367	0.004
	Secondary	−0.019	−0.053 – 0.014	1.277	0.258
Household (Ref.: alone)	With partner	0.030	−0.004 – 0.063	2.948	0.086
	with child/children and/or another family member	0.013	−0.032 – 0.057	0.306	0.580
Physician as a member of family or friends (Ref.: yes)	No	0.025	−0.008 – 0.057	2.271	0.132
Localization of cancer (Ref.: breast)	Head and neck	−0.018	−0.099 – 0.063	0.194	0.660
	Upper digestive system	0.054	−0.024 – 0.131	1.850	0.174
	Lower digestive system	0.070	0.020–0.120	7.549	0.006
	Lung	0.040	−0.005 – 0.084	3.017	0.082
	Gynecological	−0.060	−0.146 – 0.026	1.891	0.169
	Prostate	−0.029	−0.094 – 0.036	0.772	0.380

AIC = 736.17; maximum likelihood estimation chi^2^ = 36.58; link function: identity; *p* < 0.001.

The need score for psycho-emotional support was significantly higher among patients with higher educational level than lower educational level (*p* < 0.001) and patients diagnosed with upper digestive system cancer in comparison with breast cancer patients (*p* = 0.01) (Table 9 and Figure 2).

**Table 9:** Generalized linear models (GLZ) to explain the relationship between the needs for a psycho-emotional support and a set of independent variables.

Effect	Level of effect	Parameter	95% CI of parameter	Wald function	*p*
Age		−0.002	−0.005 – 0.001	2.073	0.150
Educational level (Ref.: high)	Primary	0.017	−0.025 – 0.059	0.643	0.423
	Secondary	−0.052	−0.082 – − 0.021	11.006	<0.001
Place of residence (Ref.: village)	City	−0.016	−0.039 – 0.006	2.044	0.153
Employment (Ref.: pensioner)	Employed	−0.011	−0.061 – 0.038	0.190	0.663
	Unemployed	−0.060	−0.135 – 0.016	2.371	0.124
Marital status (Ref.: single)	Married	0.019	−0.004 – 0.042	2.649	0.104
Localization of cancer (Ref.: breast)	Head and neck	−0.046	−0.121 – 0.028	1.474	0.225
	Upper digestive system	0.090	0.019–0.161	6.192	0.013
	Lower digestive system	0.032	−0.014 – 0.078	1.874	0.171
	Lung	0.039	−0.002 – 0.079	3.419	0.064
	Gynecological	−0.025	−0.104 – 0.054	0.396	0.529
	Prostate	−0.045	−0.104 – 0.014	2.201	0.138

AIC = 569.31; maximum likelihood estimation chi^2^ = 37.01; link function: identity; *p* < 0.001.

## 4 Discussion

Cancer patients experience a wide range of unmet supportive care needs (Okediji et al., 2017; Evans Webb et al., 2021). Multiple areas of non-medical needs have been identified: informational, health care organizational, psychological, spiritual, relational, needs associated with daily living, sexuality, social and material (Okediji et al., 2017; Evans Webb et al., 2021; Bonacchi et al., 2016; Driessen et al., 2023; Wang et al., 2018; Hart et al., 2022). Identification of unmet non-medical needs, as well as characteristics of patients with the greatest unmet needs, is important to support patient wellbeing. Deficits in meeting cancer patients’ needs could lead to dissatisfaction with the health care system and reduce patients’ quality of life (Hack et al., 2010; Fitch and Maamoun, 2016; Park and Hwang, 2012; Cochrane et al., 2022; Jagatap et al., 2020). However, the assessment of supportive care needs is difficult because they are subjective and the importance of each need is different for each individual patient. Unmet needs of cancer patients have been measured using a wide variety of instruments, with different scales, and various psychometric properties (Rimmer et al., 2022; Wen and Gustafson, 2004). Some studies were focused on selected domains such as information needs (Neumann et al., 2011; Ha and Longnecker, 2010; Jenkins et al., 2001; Meredith et al., 1996; Alamanou et al., 2016; Leydon et al., 2000; Hsieh et al., 2018) or psychological and physical needs (Amenu et al., 2023).

Some socio-psycho-demographic factors could influence self-reported needs: age, sex, marital status, educational level, income, receiving relational/psychological support from family/friends, having a physician among close family/friend and clinical factors such as cancer localization, clinical stage of disease, intent for treat (Okediji et al., 2017; Puts et al., 2012; Hart et al., 2022; Gebresillassie et al., 2021). According to Granular Interaction Thinking Theory posits that macro-level order emerges from structured micro-level interactions (Nguyen et al., 2025). Small elements can affect the whole. Based on this theory many minor single factors could affect a patient’s whole well-being. It seems to be necessary to try finding those small things. Unmet needs may be among those elements. There are some studies concerning the unmet supportive care needs in patients diagnosed with only a particular type of cancer: breast and gynecological cancer (Graf et al., 2020; Uslu-Sahan and Gulcan, 2023; von Heymann-Horan et al., 2013), colorectal cancer (Sodergren et al., 2019), lung cancer (Giuliani et al., 2016; Hsieh et al., 2018; Sanders et al., 2010; Li and Girgis, 2006; Yang et al., 2023). Some studies only included patients in certain stage of disease: newly diagnosed (Puts et al., 2012), early stage (Harrison et al., 2009; Okediji et al., 2017), advanced stage (Rimmer et al., 2022; Driessen et al., 2023; Wang et al., 2018; Hart et al., 2022). Some studies were carried out among young patients (Okamura et al., 2021), others among older patients (Puts et al., 2012). It appears that type of cancer, stage of disease and patient’s age could have an impact on results of these studies. Moreover cultural background may have a potential impact on self-reporting of unmet needs by cancer patients. When interpreting the results of studies reported in particularly countries it is important to consider differences in population culture (or possibly mind-set), as well as the organization of the health care system, priorities in health care and economic disparities: Germany (Graf et al., 2020), Scotland (Meredith et al., 1996), UK (Evans Webb et al., 2021; Ha and Longnecker, 2010; Sodergren et al., 2019; Jenkins et al., 2001), US (Neumann et al., 2011), Greece (Alamanou et al., 2016), Nigeria (Okediji et al., 2017), Italy (Tamburini et al., 2003; Bonacchi et al., 2019; Bonacchi et al., 2016; Bonacchi et al., 2018; Chiesi et al., 2017), Denmark (Sodergren et al., 2019), Japan (Okamura et al., 2021), Netherland (Driessen et al., 2023), Turkey (Erdoğan Yüce et al., 2021), Ethiopia (Amenu et al., 2023; Gebresillassie et al., 2021; Amane et al., 2021), Taiwan (Hsieh et al., 2018), Canada (Puts et al., 2012; Giuliani et al., 2016), Australia (Sanson-Fisher et al., 2000; Hart et al., 2022), Hong Kong (Wang et al., 2018), China (Yang et al., 2023).

In Poland there is a lack of studies concerning supportive care needs among cancer patients. In the current study unmet non-medical needs were assessed in a large sample (n = 1,062) of cancer patients. The study included patients diagnosed with different types of cancers. In the analysis different socio-demographic factors were taken into account. The instrument which was chosen to determine the supportive care needs was the NEQ (Tamburini et al., 2003). The heterogeneity of survey methodology used in previous studies has made it difficult to position the results of this analysis in the broader context of published literature. Cancer patients in current study expressed some unmet supportive care needs – the mean total NEQ score was 0.44 (on scale 0–1). In general, higher levels of unmet needs were reported by younger patients, those living with a partner and patients diagnosed with digestive system cancers and lung cancer. Bonacchi et al. (2019), using the same instrument (NEQ) to assess unmet needs among Italian cancer patients, reported a lower total NEQ score than in current analysis (0.30 vs. 0.44). It was shown that Italian cancer patients more frequently reported perceived unmet needs if they had a lower education level and were treated at in-patient clinics (Bonacchi et al., 2019). However, there were no significant differences in self-reported unmet needs by patients associated with age, marital status or cancer localization. In a study among Ethiopian cancer patients, Gebresillassie et al. (2021) and Turkish cancer patients Uslu-Sahan and Gulcan (2023) determined that sex and residence were independent predicting factors for unmet supportive care needs.

In a Danish study (von Heymann-Horan et al., 2013) 41% of 261 breast cancer patients expressed unmet needs. Women, who were younger, single or had a higher level of education were more likely to have unmet supportive care needs (von Heymann-Horan et al., 2013). Giuliani et al. (2016) observed that age and tumor-related factors were associated with supportive care needs among lung cancer patients. Younger age was determined as a factor increasing the tendency to have more unmet needs (Abdollahzadeh et al., 2014).

In the current study the subtotal NEQ score was the highest for informative needs (mean score 0.55). Informative needs included seeking more information about diagnosis, examinations, treatments, future prognosis, and better dialogue with clinicians. The subtotal NEQ score for material needs was also relatively high (mean score 0.49). Material needs included the need for more information about economic insurance in relation to illness, economic help and better services from the hospital (bathrooms, meals, cleaning). Informative needs and material needs were also more frequently reported among Italian cancer patients (mean scores: 0.35–0.48 and 0.28–0.29, respectively) (Bonacchi et al., 2019; Bonacchi et al., 2016; Bonacchi et al., 2018). However, Italian cancer patients in general expressed lower levels of unmet needs in various domains than patients in current study conducted using the same tool. In some studies (Neumann et al., 2011; Ha and Longnecker, 2010; Jenkins et al., 2001; Meredith et al., 1996; Alamanou et al., 2016; Gebresillassie et al., 2021; von Heymann-Horan et al., 2013) cancer patients had the highest unmet supportive care needs in the domains financial and health system and information. Reviews (Okediji et al., 2017; Evans Webb et al., 2021) confirmed that the health system and information domain is the most reported by cancer patients. Patients wished to know what treatment they would receive, why it was chosen, how it worked, what they could expect from therapy, what the possible side effects could be and how they could be relieved. Patients needed more information about their diagnosis, prognosis and the chance of a relapse (Neumann et al., 2011; Ha and Longnecker, 2010; Jenkins et al., 2001; Meredith et al., 1996; Alamanou et al., 2016). In a small, single center Polish study conducted among cancer patients in a hospice showed that informative needs were most frequently expressed (Włostowska et al., 2018). According to a novel theory of information processing in the human mind – Mindsponge, the information cannot be absorbed, it cannot be processed and stored in the mind, and affect the person’s subsequent feeing, thoughts, and behaviors (Vuong, 2023). Cancer-related information could results in patients’ reaction on diagnosis, compliance with treatment and thinking about the future. Therefore, it is not surprising that patients pay special attention to information needs. Informative needs were most frequently expressed at the beginning of the disease and at the time of recurrence/progression (Akechi et al., 2011; Puts et al., 2012; Hsieh et al., 2018; Bonacchi et al., 2018). In the current study there was no significant correlation between time from cancer diagnosis and informative needs. In other studies, the need for more information about disease, treatment etc. were expressed predominantly by patients with breast cancer and hematologic cancer (Alamanou et al., 2016), younger patients (Sanson-Fisher et al., 2000; Evans Webb et al., 2021; Puts et al., 2012; Ha and Longnecker, 2010; Jenkins et al., 2001; Meredith et al., 1996; Alamanou et al., 2016), women (Puts et al., 2012; Meredith et al., 1996), and those with a higher education level (Alamanou et al., 2016). However, Bonacchi et al. (2019) using the same questionnaire (NEQ) did not show differences between informative needs due to age, sex, marital status and educational level. Giuliani et al. (2016) did not observed relationships between supportive care needs in the information domain and age, sex or level of education, marital status or number of household members among lung cancer patients. In the current study sex and education level have not been shown to have a statistically significant influence on informative needs. Whereas the informative needs score was significantly higher among patients who were younger, married or diagnosed with upper digestive system cancer.

The need least expressed by patients in the current analysis was for psycho-emotional support (contact with a psychologist, priest or patients with similar experiences). Some studies indicated that health system, information and psychologic domains of needs were reported at similar high levels to each other (Sanson-Fisher et al., 2000; Puts et al., 2012; Lewandowska et al., 2020b). Other studies indicated psychological needs as most frequently perceived by cancer patients (Driessen et al., 2023; Amenu et al., 2023; Giuliani et al., 2016; Okamura et al., 2021; Sodergren et al., 2019; Amane et al., 2021). Amane et al. (2021) reported that old age was significantly associated with unmet psychological needs among cancer patients. Whereas another study showed in contrast that younger patients were more frequently seeking psychological support (Sanson-Fisher et al., 2000). In the current study age was not reported as an independent predictor of unmet psycho-emotional needs. There were also no differences according to gender. However, in some studies females were more often seeking psychological support (Sanson-Fisher et al., 2000; Giuliani et al., 2016). In the current study there was a significant higher psycho-emotional needs score among patients with a higher education level and those diagnosed with upper digestive system cancer.

The type of cancer had a significant impact on both total and subtotal NEQ scores. Total NEQ score was the highest among patients with upper digestive system cancer and lowest among breast cancer patients. The highest subtotal NEQ score was noted in the case of informative needs for all types of cancers (except gynecological cancer patients: the NEQ score was equal for informative and material needs). Patients with upper digestive system cancer had especial supportive care needs in the information domain. Whereas head and neck, and breast cancer patients expressed the lowest informative needs scores. The same situation was observed in the case of needs related to assistance/care and needs for psycho-emotional support. However, these scores were twice lower than informative needs scores. Relational needs were especially important for patients with lower and upper digestive system cancers as well as lung cancer. Whereas breast cancer patients expressed the lowest relational needs score. Among all unmet non-medical needs, seeking material support was relatively frequently expressed. The highest material needs score was observed in the case of patients with lower digestive system cancer and the lowest in prostate cancer patients. Similar results of high NEQ scores among patients with upper digestive system cancer (pancreas and stomach) were presented by Bonacchi et al. (2019). The authors (Bonacchi et al., 2019) also observed high NEQ score among breast cancer patients but in the current study women with breast cancer did not express as much needs for supportive care. Other authors investigating patients with different cancers showed that breast cancer patients reported the lowest level of unmet supportive care needs (Sanson-Fisher et al., 2000). Some authors indicated the relatively high level of supportive care needs across a range of domains among lung cancer patients (Giuliani et al., 2016; Li and Girgis, 2006).

Breast cancer is a particularly well publicized type of cancer; there are a lot of brochures, information in media and instances where some breast cancer patients share their experiences, etc. This could explain why breast cancer patients have less unmet supportive care needs. Upper digestive system and lung cancer are diseases with a worse prognosis and that may cause more concerns; patients seldom share their experiences and they are under-represented in social media.

### 4.1 Study limitations

In the current study there were no analyses of anxiety, distress or quality of life among respondents and no mental health measurements were provided. Data on clinical stage or the intention of treatment, which may be potentially associated with the unmet needs of cancer patients, were not collected.

### 4.2 Study implications

Identification of cancer patients’ unmet non-medical needs should be a part of daily clinical practice. Therefore, an appropriate tool to recognize the patients’ needs should be used. It could help to provide appropriate support to each individual patient.

## 5 Conclusion

Cancer patients expressed some unmet supportive care needs. It is crucial for improve patient-medical staff communication to investigate what kind of non-medical needs are most important for cancer patients. It seems that meeting unmet informative needs is especially desirable. Identifying groups of patients with high rates of unmet supportive care needs will be useful to find a target for an adequate support. In the current study younger respondents, living with a partner and patients suffering from digestive system cancer or lung cancer seem to require special attention. Younger age was a predictor of unmet informative needs, needs related to assistance/care and material needs. Lower education had an impact on unmet assistance/care and relational needs whereas higher education was related with unmet psycho-emotional needs. Marriage patients desired more information, men - more assistance/care support, pensioners – more material support. Cancer type was a significant factor affecting different domains of needs. Further investigations concerning non-medical needs among cancer patients, especially informative needs are needed.

## Data Availability

The raw data supporting the conclusions of this article will be made available by the authors, without undue reservation.
